# Dietary Patterns in Portuguese Children and Adolescent Population: The UPPER Project

**DOI:** 10.3390/nu13113851

**Published:** 2021-10-28

**Authors:** Milena Miranda de Moraes, Bruno Oliveira, Cláudia Afonso, Cristina Santos, Duarte Torres, Carla Lopes, Renata Costa de Miranda, Fernanda Rauber, Luiza Antoniazzi, Renata Bertazzi Levy, Sara Rodrigues

**Affiliations:** 1 Faculty of Nutrition and Food Sciences, University of Porto, 4150-180 Porto, Portugal; bmpmo@fcna.up.pt (B.O.); claudiaafonso@fcna.up.pt (C.A.); cristinasantos@fcna.up.pt (C.S.); dupamato@fcna.up.pt (D.T.); saraspr@fcna.up.pt (S.R.); 2 Associated Laboratory ITR, Laboratory for Integrative and Translational Research in Population Health, Institute of Public Health, University of Porto, 4050-600 Porto, Portugal; carlal@med.up.pt; 3 Artificial Intelligence and Decision Support (LIAAD), Institute for Systems and Computer Engineering, Technology and Science (INESC TEC), 4200-465 Porto, Portugal; 4 Center for Health Technology and Services Research (CINTESIS), Associate Laboratory RISE-Health Research Network, University of Porto, 4200-450 Porto, Portugal; 5 Faculty of Medicine, University of Porto, 4200-319 Porto, Portugal; 6 Center for Epidemiological Research in Nutrition and Health, University of São Paulo, São Paulo 01246-90, Brazil; renata.miranda@uftm.edu.br (R.C.d.M.); rauber@usp.br (F.R.); luiza.antoniazzi@hotmail.com (L.A.); rlevy@usp.br (R.B.L.); 7 Department of Nutrition, Institute of Health Sciences, Federal University of Triângulo Mineiro, Uberaba 38025-440, Brazil; 8 Department of Preventive Medicine, Faculty of Medicine, University of São Paulo, São Paulo 01246-90, Brazil; 9 Department of Nutrition, School of Public Health, University of São Paulo, São Paulo 01246-90, Brazil

**Keywords:** dietary patterns, latent class analysis, ultra-processed foods, diet quality, feeding behaviour

## Abstract

Considering the nature, extent, and purpose of food processing, this study aims to identify dietary patterns (DPs) and their associations with sociodemographic factors and diet quality in Portuguese children and adolescents. Cross-sectional data were obtained from the National Food, Nutrition and Physical Activity Survey (2015–2016) of the Portuguese population. Dietary intake was obtained from two non-consecutive days and food items were classified according to the NOVA system. The proportion (in grams) of foods in the total daily diet was considered to identify DPs by latent class analysis, with age and sex as concomitant variables. Associations of DPs with sociodemographic characteristics were assessed using multinomial logistic regression. Linear regressions adjusted by sociodemographic characteristics tested associations of DPs with diet quality. DPs identified were: “Unhealthy” (higher sugar-sweetened beverages, industrial breads, and sausages intake), “Traditional” (higher vegetables, fish, olive oil, breads, ultra-processed yogurts, and sausages intake), and “Dairy” (higher intake of milk, yogurt, and milk-based beverages). “Unhealthy” was associated with older ages and lower intake of dietary fibre and vitamins and the highest free sugars and ultra-processed foods (UPF), although all DPs presented significant consumption of UPF. These findings should be considered for the design of food-based interventions and school-feeding policies in Portugal.

## 1. Introduction

Childhood and adolescence are important stages of growth and development, which demand special energy and nutrient requirements, representing a phase of potential nutritional risk. Food consumption in this age group is usually characterized by fast foods, snacks and sugar-sweetened beverages, insufficient fibre intake, and high intake of saturated fats and sodium [1,2]. Dietary intake of preschool children in European Mediterranean countries is also known to present excessive sodium intake with high frequency of fruit and vegetables, but also sugar-sweetened beverages and snacks, mostly ready-to-eat [3]. In Portugal, children and adolescents presented a high prevalence of inadequacy of saturated fat, fibre, sugar, and salt. In terms of food consumption, these age groups had a higher prevalence of inadequate intake of fruits and vegetables than adults and elderly, and adolescents had the highest consumption of sugar-sweetened beverages and soft drinks [4].

As a consequence of advances in the food production systems and social and lifestyle trends, the intake of fat and sugars, and consumption of ready-to-eat products have been increasing worldwide, reflecting changes in the populations’ dietary pattern worldwide, including Portugal [5,6]. In this context, a new classification system named NOVA [7] has been proposed to categorize foods according to the nature, extent, and purpose of industrial processing into four groups: unprocessed or minimally processed foods, processed culinary ingredients, processed foods, and ultra-processed foods (UPF). According to the NOVA classification system, UPF are ‘formulations of ingredients, mostly for industrial use only, derived from a series of industrial processes’ [7] and their use in dietary intake assessment has been increasing worldwide. 

UPF consumption has been related to several adverse health outcomes, such as overweight/obesity, abdominal obesity, dyslipidaemia, metabolic syndrome, depression, cardiovascular and cerebrovascular disease, as well as total mortality, in adults [8]. Among children and adolescents, UPF consumption has been associated with some outcomes such as increased body fatness [9], the occurrence of asthma or wheezing [10], and changes in serum lipid profile [11]. These associations may be explained by the impact of ultra-processed foods on diet quality, which has been seen among Portuguese adults and the elderly population [12] and also in other national representative samples which included children and adolescents [13,14,15,16,17]. 

With regard to diet quality evaluation, dietary pattern analyses represent another approach that allow diets to be described as a whole and are broadly used to investigate the role of social, demographic, and economic factors in the adherence to a certain dietary pattern, as well as its relationship with health behaviours and adverse outcomes. Dietary patterns can be assessed by score-based approaches (a priori) or using data-driven techniques (a posteriori) [18]. A few studies have been conducted aiming at describe a posteriori dietary patterns of Portuguese children and adolescents, either to relate to social and behavioural factors [19] or to anthropometric and metabolic health status [20]. However, there is still lack of evidence about dietary pattern analyses that consider the nature, extent, and purpose of food processing.

Dietary behaviours established in childhood may significantly track into adolescence [21] and adulthood [22] and can be reflected in adverse health outcomes such as overweight [23], increased adiposity [24], hypertension [25], diabetes, and metabolic syndrome [26] across the lifespan. This highlights the importance of identifying unhealthy dietary patterns in children and adolescents to prevent potential harms to health, identify axes of action for public policies, and improve the development of interventions adapted to the needs of these age ranges. This study aims to identify dietary patterns using food groups based on the NOVA classification system and their associations with socioeconomic, demographic, and nutritional factors in Portuguese children and adolescents.

## 2. Materials and Methods

### 2.1. Study Design and Subjects

The UPPER project uses data from the National Food, Nutrition and Physical Activity Survey of the Portuguese General Population aged between three months and 84 years (IAN-AF 2015–2016), on which a protocol and methodology have already been published [27,28]. The IAN-AF was conducted with a nationally representative sample that was selected by multistage sampling, using as a frame the National Heath Registry, stratified by the seven Statistical Geographic Units: North, Centre, Lisbon Metropolitan Area, Alentejo, Algarve, and the autonomous regions of Azores and Madeira. Primary health care units and individuals, according to sex and age groups, were randomly selected on each stage of sampling.

For the present study, we chose to exclude children aged less than 3 years (*n* = 806), assuming substantial differences in the diet of infants and toddlers, namely breastfeeding and the use of baby bottles and other baby foods [29]. Thus, the total sample was made up of 1153 individuals, including 521 children (3–9 years) and 632 adolescents (10–17 years) who participated in two dietary interviews.

### 2.2. Sociodemographic and Anthropometric Data Collection

The IAN-AF data collection was performed by Computer-Assisted Personal Interviewing (CAPI) face-to-face interview, using the “You eAT&Move” platform, especially developed for the survey. This platform was composed of three modules: “You”, “eAT24”, and “Move”, comprising the evaluation of several dimensions [27,28], of which the present study focuses on dietary intake and sociodemographic data. In order to lessen seasonal variability, field work lasted for 12 months, and two face-to-face interviews were performed at the participant’s home or at a health care centre with an interval of 8 to 15 days between.

Sociodemographic data were obtained, in the first interview: sex (male/female) and birth date, using IAN-AF methodology [27]. Parental educational level was defined as the maximum level of education of any of the parents and is presented into three categories: none, 1st, and 2nd cycle of primary education; 3rd cycle of primary education and high school; and higher education level.

Weight and height were evaluated according to standard procedures [30], by trained personnel. Body weight was measured to the nearest tenth of a kilogram using a digital scale (SECA 813, Hamburg, Germany) and height was measured to the nearest centimetre using a portable wall stadiometer (SECA 213, Hamburg, Germany). Body mass index—BMI (kg/m^2^)—was calculated and analysis included the classification of overweight according to the World Health Organization sex- and age-specific z-scores (z-BMI) for children [31] and adolescents [32].

### 2.3. Dietary Assessment and Food Processing Classification

Dietary intake was obtained by two non-consecutive food diaries (for children < 10 years) or by two 24 h recalls, using the eAT24 module. Food diaries were completed by a child’s parent or other main caregiver, followed by a face-to-face interview, allowing the respondent to add details related to food description and quantification. Adolescents answered the 24 h recalls, with the need for parental or caregiver help for those aged between 10 and 14 years. The eAT24 software follows the Automated Multiple-Pass Method [33] for 24 h (five steps) to obtain details about each consumed food or beverage including name, quantity, brand, and cooking methods, as well as the place and time for each eating occasion. When the weight or volume of consumed food item was unknown, food portion size was estimated with the help of an illustration book, a household measure list, and package information. The eAT24 uses the Portuguese Food Composition Table [34] to estimate energy and nutrients intake from the report of food consumption obtained on the food diaries or 24-h recalls. For those items not included in the composition table, nutritional composition was obtained from the European Food Information Resource database [35] or from the food labelling information.

All reported food and beverages that resulted from a recipe were disaggregated to the ingredient level allowing classification to the extent and purpose of food processing according to the NOVA system. NOVA system classifies foods and beverages into four groups, namely: (1) Unprocessed or minimally processed foods, which are those consumed as obtained in nature or that had undergone industrial processes that do not add any substances to the original food, such as drying, boiling, freezing or others, with the objective of extending their shelf life or making their preparation easier. Some examples of group 1 are cereals, fruits, eggs, and fresh meat; (2) Processed culinary ingredients are those obtained directly from group 1 foods or from nature which are used in the preparation, seasoning, and cooking of foods, like oils and fats, sugar and salt; (3) Processed foods, which are industrial products composed by adding a substance found in group 2 to group 1 foods, usually to increase their durability, and that can include cooking methods, for example canned or bottled vegetables, fruits and legumes, salted, cured, or smoked meats or fish; (4) Ultra-processed foods are formulations of ingredients, mostly of exclusive industrial use, that result from several industrial processes and frequently have added colours, flavours, emulsifiers, and other cosmetic or sensory intensifying additives to make the final product palatable. Some examples of group 4 products are soft drinks, confectionery, sausage, and other reconstituted meat products [7].

Two experts in food consumption assessment and in the NOVA system independently conducted the classification of 1778 food items. Afterward, another expert researcher checked the classifications, pinpointing discrepant items to be discussed among all researchers, who performed the classification by consensus. In case of dubious classification, experts decided on the most conservative one. 

We also calculated the average dietary content of total energy as well as macro- and micronutrients for the whole sample, in order to identify differences among dietary patterns (DPs). Total energy intake was expressed as kcal/day, and dietary energy density was obtained by dividing the total energy by the total amount consumed, in grams. Dietary content of proteins, carbohydrates, free sugars, total fats, and saturated fats were expressed as a percentage of total energy intake, while dietary content of fibre, vitamins and minerals were expressed as nutrient density (grams, milligrams or micrograms per 1000 kcal). The content of free sugars was estimated using a specific algorithm [36] and previously applied in the IAN-AF 2015–2016, as described elsewhere [37].

### 2.4. Dietary Patterns Analyses

All reported food items classified according to the NOVA system were divided into 42 food subgroups, of which contribution in grams (% of grams related to total grams consumed in 24 h) were considered to obtain dietary patterns. To minimize the impact of zero inflations and noncontinuous variables from the food diaries and 24-h recalls, each food subgroup was divided into categories of consumption, according to the percentage of zeros: food subgroups that presented less than 20% of zeros were categorized in terciles; food subgroups that had more than 20% but less than 80% of zeros were also divided into three categories—no consumption, below consumers median, above consumers median; lastly, food subgroups that presented more than 80% of zeros were separated in a dichotomous variable, whether subjects consumed or not. 

Dietary patterns (DP) were derived a posteriori by a latent class analysis model including sex and age as concomitant variables. This is a person-centred approach, which identifies mutually exclusively subgroups of individuals (in categories) with similar dietary patterns [38]. Latent class analysis for DP identification was conducted using a polytomous outcome variables (poLCA) package for the R language and software environment for statistical computation (version 4.0.3, R Foundation for Statistic Computing, Austria, 2020). Models with two to nine latent classes were identified. The number of selected classes (patterns) was decided based on the lower value of the Bayesian Information Criterion (BIC) and substantive interpretation. Subjects were assigned to each pattern according to the highest probability of class membership, and selected DP were then characterized using weighted prevalence of individuals on extreme categories of consumption of food subgroups for each dietary pattern.

### 2.5. Statistical Analyses

Logistic regression analysis was performed to associate the highest category of consumption of subgroups with each dietary pattern membership. Multinomial logistic regression bivariate and multivariate models were performed to obtain crude and adjusted odds ratios (OR) and respective 95% confidence intervals (CI) were used for the association of dietary patterns with sex, age, geographical region, parental educational level, and z-BMI. In addition, bivariate and multivariate linear regression models were performed to test for differences in energy and nutrients intake across dietary patterns, with Sidak adjustment for multiple comparisons. All statistical analyses were performed on SPSS statistical software package version 27 (SPSS Inc., Chicago, IL, USA) using complex sample analyses, to consider the study design effect. A significance level of 5% was adopted in all analyses.

## 3. Results

### 3.1. Dietary Patterns

**Figure 1:**
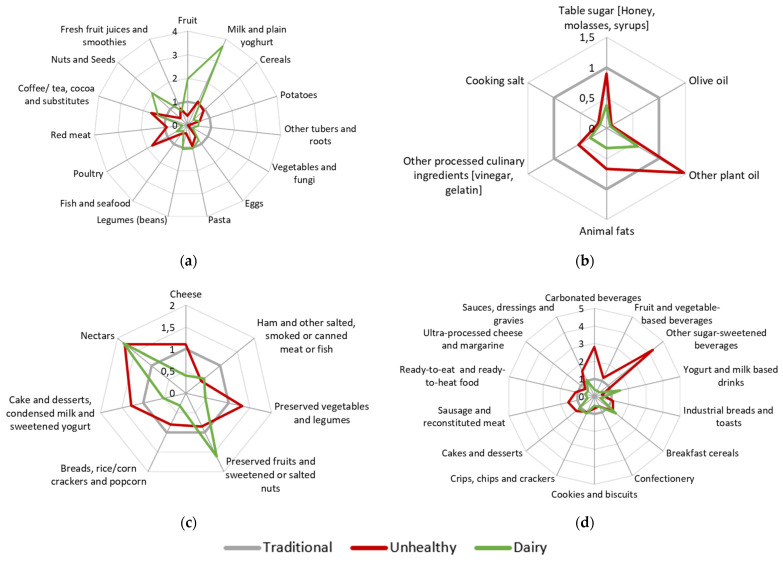
Odds ratio of being in the highest category of consumption of NOVA food subgroups for “Traditional”, “Unhealthy”, and “Dairy” DPs: (**a**) Unprocessed or minimally processed food; (**b**) Processed culinary ingredients; (**c**) Processed food; (**d**) Ultra-processed food. Traditional DP was used as reference.

The latent class model with sex and age as concomitant variables extracted three DPs (two classes, BIC = 89,704.02; three classes, BIC = 89,046.64; four classes, BIC = 89,493.7). Table 1 presents the proportion of subjects within extreme categories of consumption of food subgroups based on NOVA classification. The first dietary pattern (DP1) was followed by 51.1% of subjects and had a higher consumption of items from NOVA group 4, as sugar-sweetened beverages, industrial breads, and sausages, and lowest consumption of group 1 items, as fresh fruits, vegetables, and legumes, being labelled as “Unhealthy”. DP2 was labelled as “Traditional” and followed by 36.2% of subjects, with highest consumption of some food items from NOVA groups 1 (vegetables and fish), 2 (olive oil and cooking salt) and 3 (breads), but also high consumption of some items from group 4 foods (ultra-processed yogurts, industrial breads, and sausages). Finally, 12.7% of subjects followed DP3 labelled “Dairy”, which had the highest consumption of milk and plain yogurt, and milk-based beverages (from NOVA groups 1 or 4, respectively) and also the lowest consumption of most NOVA group 3 items, likewise some other NOVA 4 group subgroups, such as industrial breads and sausages and other reconstituted meat products. Figure 1 represents the odds ratio of being in the highest category of consumption of NOVA food subgroups for each DP. Graphical representations clearly show that the three observed patterns are visually different in the four NOVA categories. In detail, subjects following “Unhealthy” DP presented three times higher odds of being in the highest category of consumption for carbonated beverages and over four times higher odds of presenting high consumption of other sugar-sweetened beverages than those following “Traditional” DP.

### 3.2. Socioeconomic Characteristics and Nutritional Factors According to Dietary Patterns

The socioeconomic and demographic characteristics and nutritional status of subjects according to their DPs are shown in Table 2. Subjects who followed the “Unhealthy” DP presented the lowest prevalence of parental higher education. “Dairy” DP had no adolescents and presented the lowest age mean (5.7 years), compared to “Traditional” (9.8 years) and “Unhealthy” (12.1 years). Models expressing dietary patterns’ relations with sociodemographic factors and body mass index are described in Table 3. Adjusted multinomial logistic analysis showed “Unhealthy” DP was positively associated with age (OR = 1.17; 95% CI 1.09–1.23), while “Dairy” DP was negatively associated with age (OR = 0.66; 95% CI 0.61–0.72).

Traditional DP was used as reference and is not shown in order to avoid redundancy. “Traditional” DP had higher consumption of vegetables, fish, and seafood, as well as olive oil and cooking salt; “Unhealthy” DP was especially marked by a high consumption of soft drinks and other sugar-sweetened beverages; “Dairy” DP presented greater intake of milk and plain yogurt, but also ultra-processed milk-based drinks.

Nutritional intake of subjects following each DP is presented on Table 4. Data are presented as crude and adjusted means for the “Traditional” DP, which was used as reference, besides crude and adjusted coefficients for “Unhealthy” and “Dairy” DPs. Therefore, crude and adjusted means for both “Unhealthy” and “Dairy” DPs can be obtained by adding their respective coefficients to the “Traditional” DP means (for example, the crude mean for total energy on the “Unhealthy” DP was 1959.36 kcal, which corresponds to the “Traditional” DP mean plus the crude coefficient of +124.67). After adjusting for sociodemographic variables, individuals in the “Traditional” pattern reported statistically significant lower intake of carbohydrates and higher intake of processed culinary ingredients and total fats compared to those in the other patterns. Followers of “Unhealthy” DP reported significantly higher intake on ultra-processed foods and free sugars, and lower intake on unprocessed and minimally processed foods, dietary fibre, vitamin A, vitamin C, folates, sodium, potassium, magnesium, and iron, than those following “Traditional” DP. Lastly, subjects following the “Dairy” DP showed lower total energy and energy density, while higher intake of potassium, calcium, phosphorus, magnesium, and zinc, compared to “Traditional” DP.

## 4. Discussion

In this study, we were able to identify three DPs in a national representative sample of Portuguese children and adolescents, using food items classified based on NO-VA system: (1) The “Unhealthy” DP was especially marked by a high consumption of soft drinks and other sugar-sweetened beverages, (2) “Traditional” DP had higher consumption of vegetables, fish and seafood, as well as olive oil and cooking salt; (3) “Dairy” DP presented greater intake on milk and plain yogurt, but also ultra-processed milk based drinks. 

Since dietary intake may be assessed through different methods, as well as the extraction of dietary patterns can be performed by some different statistical approach-es, the comparison of our findings with other studies is here presented based on the subjects’ age range. In general, extracted DPs in studies conducted with children and adolescents also include a traditional (also named “healthy” or “prudent”) and an un-healthy (also named as “processed”, “western” or “energy-dense”) DP [19,20,39,40], as seen in our study. Nevertheless, only in a Portuguese population-based sample of 13 years-old adolescents, a DP marked exclusively by high consumption of dairy products was also observed [19]. 

The descriptive analysis of sociodemographic characteristics and BMI according to DP has shown mean age was lower in “Dairy” DP, followed by “Traditional” and “Unhealthy” DP, and this association was confirmed with higher odds of being in “Unhealthy” DP and lower odds of being in “Dairy” DP by age, compared to those on “Traditional” DP, even after adjustments. A higher percentage of older children from European countries were also allocated in a “sweet and processed” DP [39], while a higher proportion of Spanish adolescents was observed in a “health conscious” DP [40]. 

Subjects in the “Unhealthy” DP presented the lowest frequency of parents with higher educational level. However, this expected inverse association, which was seen in children and adolescents from other High Human Development countries [41], was not confirmed in our multinomial logistic analysis, after adjustment for other socio-demographic variables and BMI. Subjects living in Azores presented higher odds of following “Dairy” DP compared to the North, which was expected since dairy consumption in this region is the highest observed in Portugal [4] and the dairy sector has a major importance to the production and economy of that archipelago [42]. On the other hand, living in Lisbon Metropolitan Area was positively associated with the “Unhealthy” DP, which converges with some unfavourable health characteristics observed for this region, such as lower prevalence of physical activity and lower adherence to a Mediterranean dietary pattern, and higher consumption of red meat and time spent on sedentary behaviours [4].

Regarding energy and nutrient intake, in comparison to “Traditional” DP, “Un-healthy” DP was higher on ultra-processed foods, free sugars and carbohydrates, and lower in total fats, dietary fibre, vitamin A, vitamin C, folates, sodium, potassium, magnesium and iron, while “Dairy” DP showed lower total energy, energy density, processed culinary ingredients and total fats, and higher intake of carbohydrates, potassium, calcium, phosphorus, magnesium and zinc, compared to “Traditional” DP. The findings about “Unhealthy” DP are supported by other studies on which an un-healthy DP [43] or a higher consumption of UPF [15,17,44] were related with lower nutritional quality of diets. However, the dietary intake of sodium in our study was lower for those following the “Unhealthy” DP, oppositely to other findings of studies on UPF consumption [14,15], but in accordance with observations from Brazil [45], while no as-sociation was observed in the USA [46], all of them using the NOVA system. This may be because the main sources of sodium in these countries are not UPF. In the Portuguese diet, sodium intake was mainly from the added salt of culinary preparation, summing up more than half of total sodium intake (unprocessed or minimally processed foods plus processed culinary ingredients), followed by processed foods [12]. In addition, it can be highlighted that the “Traditional” DP was associated with some UPF that have higher sodium, as milk-based drinks, sausages and meat products and industrial breads and toasts.

To our knowledge, this is the first study to perform dietary pattern analysis using food groups based on the extent and purpose of food processing in children and adolescents. In Lebanese adults, a previous investigation of dietary pattern considering the NOVA classification resulted in two main patterns primarily defined by NOVA groups - the ‘ultra-processed’ and ‘minimally processed/processed’ - and a higher adherence to the last one was significantly associated with lower odds for metabolic syndrome, hyperglycemia and low HDL-C level [47]. In our study, “Unhealthy” DP presented the highest intake of UPF, expressed in percentage of total energy. However, even “Traditional” and “Dairy” DPs had more than 30% of total energy intake from UPF, which demonstrates how the consumption of ultra-processed foods is relevant among Portuguese children and adolescents, regardless of their eating habits. 

In the present study, there was no relationship between extracted DPs and BMI. The associations of DPs with the risk of obesity in children and adolescents could not be confirmed in many studies included in a systematic review on this topic [48]. In the same way, there is no consensus on the association between DPs and cardiometabolic risk in children and adolescents [40,49,50]. However, there is evidence supporting the tracking of DPs established on childhood through adulthood [22] which can also represent a higher risk of noncommunicable diseases later in life [51,52] and highlights the importance of studying DPs in this age group.

This study had some limitations and strengths. Dietary patterns were extracted on the basis of food consumption information obtained from two non-consecutive days. Although it is known that, ideally, food consumption-based studies should consider longer reporting periods, we had an adequate distribution of weekdays, so that the eating pattern of the weekend could be taken into consideration [28]. Regarding dietary assessment methods used in this study, food diaries have the limitation to rely on respondent’s literacy and ability to describe portion sizes, but face-to-face interviews were performed with the research assistants to review diaries in order to overcome this limitation. Also, dietary intake of adolescents was estimated by 24-h recalls, an instrument that depends on the respondent’s memory, but the multiple-pass method has been shown to be accurate [33] and photographs of different portions were used to minimize difficulties in quantifying consumed foods and the omission of possible for-gotten ones. Moreover, fieldwork was conducted within 12 months, in order to account for seasonal variability. Another strength of this study is that its data were collected in the latest national representative survey on food, nutrition and physical activity, al-lowing findings in this sample to reflect Portuguese children and adolescents. Additionally, the cross-sectional design makes it impossible to establish causal inferences, since DPs and its possible outcomes were observed at the same time. However, this is a pioneering study in these age groups and we believe the fact of using food groups based on NOVA classification system to extract dietary patterns is another strength of our study, since this approach made it possible to obtain dietary patterns based on both the type of food (fruits, vegetables, meats) and their degree of processing, what can be used for application in nutritional interventions.

## 5. Conclusions

About half of Portuguese children and adolescents were classified in the “Unhealthy” dietary pattern, which was especially characterized by lower consumption of fruits, vegetables, and legumes, and a higher consumption of sugar-sweetened beverages. This pattern presented the highest intake of ultra-processed foods and free sugars, and lower intake of dietary fibre, vitamins, and minerals. Regardless of the dietary pattern followed, over one third of the calories consumed by Portuguese children and adolescents came from ultra-processed foods. These findings should be considered for the design of food education activities and to promote measures concerning food availability in the school environment in Portugal, as well as in food-based interventions promoting healthier dietary habits in these age groups.

## Figures and Tables

**Table 1:** Weighted prevalence * of subjects within consumption categories in each dietary pattern—Portuguese population aged 3–17 years: The UPPER project.

		DP 1 (Unhealthy)	DP 2 (Traditional)	DP 3 (Dairy)
Food Group	Consumption Category	*n* = 589 51.1%	*n* = 417 36.2%	*n* = 147 12.7%
Unprocessed or minimally processed foods				
Fruit	1st tercile	54.6 ^a^	29.0 ^b^	14.9 ^b^
	3rd tercile	18.4 ^a^	36.7 ^b^	53.3 ^b^
Milk and plain yoghurt	1st tercile	48.7 ^a^	47.6 ^a^	17.1 ^b^
	3rd tercile	19.7 ^a^	18.3 ^a^	45.2 ^b^
Cereals	1st tercile	37.5 ^a^	36.4 ^a^	68.6 ^b^
	3rd tercile	31.1 ^a^	32.8 ^a^	14.3 ^b^
Potatoes	1st tercile	48.6 ^a^	26.3 ^b^	21.9 ^b^
	3rd tercile	25.2 ^a^	38.3 ^b^	23.9 ^a,b^
Other tubers and roots	1st tercile	75.1 ^a^	15.1 ^b^	13.4 ^b^
	3rd tercile	4.3 ^a^	51.6 ^b^	32.2 ^b^
Vegetables and fungi	1st tercile	65.1 ^a^	1.4 ^b^	8.4 ^c^
	3rd tercile	4.6 ^a^	67.6 ^b^	33.1 ^c^
Eggs	No consumption	44.6 ^a^	34.6 ^a^	43.4 ^a^
	≥Median	24.7 ^a^	35.8 ^a^	30.7 ^a^
Pasta	No consumption	36.4 ^a^	35.8 ^a^	25.4 ^a^
	≥Median	31.5 ^a^	33.5 ^a^	34.3 ^a^
Legumes (beans)	No consumption	64.7 ^a^	43.4 ^b^	31.2 ^b^
	≥Median	14.6 ^a^	32.3 ^b^	33.4 ^b^
Fish and seafood	No consumption	61.1 ^a^	44.2 ^b^	39.8 ^b^
	≥Median	18.4 ^a^	33.1 ^b^	13.3 ^a^
Poultry	No consumption	33.4 ^a^	41.5 ^a,b^	28.1 ^b^
	≥Median	39.6 ^a^	27.2 ^a,b^	17.1 ^b^
Red meat	No consumption	21.8 ^a^	16.8 ^a^	16.0 ^a^
	≥Median	40.5 ^a^	43.1 ^a^	14.0 ^b^
Coffee/tea, cocoa, and substitutes	No consumption	75.8 ^a^	80.2 ^a^	80.4 ^a^
	≥Median	13.0 ^a^	8.3 ^a^	10.7 ^a^
Nuts and Seeds	No consumption	95.9 ^a^	90.8 ^a,b^	82.7 ^b^
	Consumption	4.1 ^a^	9.2 ^a,b^	17.3 ^b^
Fresh fruit juices and smoothies	No consumption	78.6 ^a^	69.2 ^a^	76.2 ^a^
	≥Median	10.9 ^a^	14.0 ^a^	9.6 ^a^
Processed culinary ingredients				
Table sugar [honey, molasses, syrups]	No consumption	53.1 ^a^	55.4 ^a^	60.1 ^a^
	≥Median	24.1 ^a^	26.2 ^a^	11.9 ^a^
Olive oil	1st tercile	48.5 ^a^	5.0 ^b^	34.7 ^a^
	3rd tercile	16.0 ^a^	67.2 ^b^	12.3 ^a^
Other plant oil	No consumption	29.3 ^a^	35.7 ^a^	42.8 ^a^
	≥Median	40.6 ^a^	31.8 ^a,b^	21.6 ^b^
Animal fats	No consumption	44.1 ^a^	40.9 ^a^	36.8 ^a^
	≥Median	26.3 ^a,b^	34.8 ^a^	14.7 ^b^
Other processed culinary ingredients [vinegar, gelatine]	No consumption	54.9 ^a,b^	38.3 ^a^	71.5 ^b^
	≥Median	22.5 ^a^	35.0 ^a^	14.4 ^a^
Cooking salt	1st tercile	45.5 ^a^	2.5 ^b^	47.6 ^a^
	3rd tercile	18.6 ^a^	59.5 ^b^	15.8 ^a^
Processed foods				
Cheese	No consumption	43.2 ^a^	43.1 ^a^	44.3 ^a^
	≥Median	30.9 ^a^	28.8 ^a^	13.8 ^b^
Ham and other salted, smoked or canned meat or fish	No consumption	76.6 ^a^	63.9 ^b^	73.3 ^a,b^
	≥Median	10.1 ^a^	20.3 ^b^	12.1 ^a,b^
Preserved vegetables and legumes	No consumption	33.6 ^a^	20.7 ^b^	25.1 ^a,b^
	≥Median	39.3 ^a^	32.9 ^a,b^	18.3 ^b^
Preserved fruits and sweetened or salted nuts	No consumption	91.8 ^a^	90.4 ^a^	85.3 ^a^
	Consumption	8.2 ^a^	9.6 ^a^	14.7 ^a^
Breads, rice/corn crackers, and popcorn	1st tercile	43.8 ^a^	40.4 ^a^	42.1 ^a^
	3rd tercile	28.3 ^a,b^	33.1 ^a^	13.6 ^b^
Cake and desserts, condensed milk, and sweetened yogurt	No consumption	93.0 ^a^	94.5 ^a^	97.0 ^a^
	Consumption	7.0 ^a^	5.5 ^a^	3.0 ^a^
Nectars	No consumption	77.6 ^a^	86.1 ^a^	77.3 ^a^
	Consumption	22.4 ^a^	13.9 ^a^	22.7 ^a^
Ultra-processed foods				
Carbonated beverages	No consumption	68.0 ^a^	82.9 ^b^	86.4 ^b^
	≥Median	16.4 ^a^	6.5 ^b^	2.7 ^b^
Fruit and vegetable-based beverages	No consumption	83.0 ^a^	85.2 ^a,b^	94.2 ^b^
	Consumption	17.0 ^a^	14.8 ^a,b^	5.8 ^b^
Other sugar-sweetened beverages	No consumption	44.1 ^a^	62.1 ^b^	64.8 ^b^
	≥Median	34.9 ^a^	11.2 ^b^	4.5 ^b^
Yogurt and milk-based drinks	No consumption	35.2 ^a^	23.2 ^b^	6.7 ^c^
	≥Median	29.3 ^a^	48.0 ^b^	58.3 ^b^
Industrial breads and toasts	No consumption	46.9 ^a^	45.7 ^a^	47.5 ^a^
	≥Median	32.0 ^a^	30.3 ^a^	13.0 ^b^
Breakfast and baby cereals	No consumption	47.3 ^a^	43.6 ^a^	20.9 ^b^
	≥Median	26.4 ^a^	20.9 ^a^	29.2 ^a^
Confectionery	No consumption	47.2 ^a^	40.4 ^a^	47.0 ^a^
	≥Median	22.9 ^a^	32.6 ^a^	24.0 ^a^
Cookies and biscuits/Packaged sweet snacks	No consumption	46.8 ^a^	33.3 ^a^	28.2 ^a^
	≥Median	25.6 ^a^	33.6 ^a^	20.8 ^a^
Crips, chips and crackers/Packaged savoury snacks	No consumption	88.0 ^a^	87.7 ^a^	87.1 ^a^
	Consumption	12.0 ^a^	12.3 ^a^	12.9 ^a^
Cakes and desserts	No consumption	49.6 ^a^	55.1 ^a^	39.9 ^a^
	≥Median	26.1 ^a^	21.2 ^a^	23.2 ^a^
Sausage and reconstituted meat products	1st tercile	37.5 ^a^	47.4 ^a^	65.9 ^b^
	3rd tercile	32.3 ^a^	23.7 ^a^	10.9 ^b^
Ready-to-eat and ready-to-heat food	No consumption	63.9 ^a^	73.2 ^a^	71.9 ^a^
	≥Median	18.6 ^a^	17.1 ^a^	9.9 ^a^
Ultra-processed cheese, margarine, and other spreads	No consumption	52.2 ^a^	49.9 ^a^	59.0 ^a^
	≥Median	21.8 ^a^	28.6 ^a^	15.8 ^a^
Sauces, dressings, and gravies	No consumption	65.3 ^a^	61.7 ^a^	73.5 ^a^
	≥Median	22.1 ^a^	15.0 ^a^	15.6 ^a^

Two classes, BIC = 89,704.02; three classes, BIC = 89,046.64; four classes, BIC = 89,493.7; * Intermediate categories (2nd tercile or below median) were not shown in order to avoid redundancy; Different letters indicate significant differences between dietary patterns at a significance level of 5%.

**Table 2:** Sociodemographic characteristics and BMI according to dietary patterns among Portuguese population aged 3–17: The UPPER project.

	*n*	DP 1 (Unhealthy)	DP 2 (Traditional)	DP 3 (Dairy)
		% (95% CI)	% (95% CI)	% (95% CI)
**Sex**				
Female	581	45.6 (40.9–50.4)	55.1 (49.6–60.6)	43.1 (30.8–56.3)
Male	572	54.4 (49.6–59.1)	44.9 (39.4–50.4)	56.9 (43.7–69.2)
**Age group**				
Children (3–9 years)	521	24.4 (19.8–29.7)	51.6 (45.9–57.2)	100
Adolescents (10–17 years)	632	75.6 (70.3–80.2)	48.4 (42.8–54.1)	-
Age (years)—mean (CI 95%)	-	**12.1 (11.7–12.6)**	**9.8 (9.4–10.3)**	**5.7 (5.2–6.1)**
**Region**				
North	187	30.9 (26.6–35.4)	39.8 (32.3–47.8)	33.6 (24.1–44.7)
Centre	217	18.6 (15.1–22.6)	20.3 (15.4–26.3)	19.6 (12.8–28.9)
MA Lisbon	176	31.5 (27.4–35.9)	26.5 (20.2–34.0)	30.1 (20.4–42.1)
Alentejo	112	7.1 (5.3–9.6)	4.9 (2.8–8.3)	4.3 (2.4–7.5)
Algarve	133	4.8 (3.6–6.3)	3.8 (2.5–5.9)	4.8 (2.8–8.3)
Madeira	168	3.2 (2.5–4.1)	3.0 (2.0–4.6)	3.3 (2.2–5.1)
Azores	160	4.0 (2.0–7.8)	1.7 (1.1–2.6)	4.2 (3.1–5.5)
**Typology of the neighbourhood**				
Predominantly rural area	123	8.7 (4.7–15.5)	7.7 (4.3–13.5)	9.6 (4.6–19.1)
Medium urban area	183	13.0 (7.6–21.2)	13.9 (6.8–26.3)	22. (11.5–38.0)
Predominantly urban area	847	78.3 (70.1–84.8)	78.3 (67.1–86.5)	68.4 (53.9–80.0)
**Parental education**				
None/primary education	155	15.1 (11.2–20.2)	8.7 (6.6–14.0)	12.0 (6.4–21.4)
Secondary/post-secondary education	572	54.8 (48.4–61.0)	44.6 (36.7–52.7)	38.7 (29.3–49.0)
Higher education	415	**30.1 (23.5–37.7)**	45.7 (37.4–54.3)	49.3 (38.6–60.1)
**Body mass index**				
Non-overweight	797	64.5 (58.3–70.3)	72.1 (66.4–77.1)	79.6 (69.4–87.0)
Overweight	351	35.5 (29.7–41.7)	27.9 (22.9–33.6)	20.4 (13.0–30.6)

Statistically significant differences are highlighted in bold.

**Table 3:** Multinomial logistic regression analysis of the associations between sociodemographic and nutritional characteristics with dietary patterns among Portuguese population aged 3–17: The UPPER project.

	Unhealthy DP		Dairy DP	
	Crude OR (95% CI)	Adjusted OR ^‡^ (95% CI)	Crude OR (95% CI)	Adjusted OR ^‡^ (95% CI)
**Sex**				
Female	**0.68 (0.48–0.98)**	0.69 (0.47–1.03)	0.62 (0.34–1.12)	0.74 (0.38–1.46)
Male	1	1	1	1
**Age**				
Age (years)	**1.16 (1.10–1.23)**	**1.17 (1.09–1.23)**	**0.67 (0.62–0.73)**	**0.66 (0.61–0.72)**
**Typology of the neighbourhood**				
Predominantly rural area	1.12 (0.59–2.14)	0.98 (0.49–1.98)	1.42 (0.69–2.92)	2.08 (0.95–4.57)
Medium urban area	0.93 (0.47–1.85)	0.94 (0.44–2.00)	1.81 (0.81–4.02)	0.69 (0.36–1.30)
Predominantly urban area	1	1	1	1
**Parental education**				
None/primary education	**2.36 (1.28–4.35)**	1.71 (0.89–3.29)	1.15 (0.48–2.74)	2.02 (0.73–5.57)
Secondary/post-secondary education	**1.86 (1.13–3.08)**	**1.82 (1.04–3.17)**	0.80 (0.44–1.47)	0.71 (0.38–1.32)
Higher education	1	1	1	1
**Body mass index**				
Non-overweight	1	1	1	1
Overweight	1.42 (0.99–1.56)	1.41 (0.88–2.26)	0.66 (0.36–1.24)	0.98 (0.50–1.95)

^‡^ Adjusted by all other variables in the table; Statistically significant differences are highlighted in bold.

**Table 4:** Nutritional intake according to dietary patterns derived by latent class analysis among Portuguese population aged 3–17 years: The UPPER project.

	Traditional DP		Unhealthy DP		Dairy DP	
	Mean		Regression Coefficient		Regression Coefficient	
	Crude	Adjusted ^a^	Crude	Adjusted ^a^	Crude	Adjusted ^a^
Total energy intake (kcal)	1834.69	1850.12	+124.67 **	−11.39	−349.62 *	−180.89 *
Energy density (kcal/grams)	0.92	0.91	−0.04 **	−0.03	−0.16 *	−0.18 *
Unprocessed or minimally processed foods (% kcal)	41.86	42.72	−4.15 *	−4.34 *	2.77 *	+2.64
Processed culinary ingredients (% kcal)	10.45	10.16	−2.41 *	−2.37 *	−2.90 *	−2.46 *
Processed foods (% kcal)	13.50	13.98	+2.03 *	+1.77	−0.94	−0.37
Ultra-processed foods (% kcal)	34.19	33.14	+4.53 *	+4.94 *	+1.06	+0.19
Proteins (% of total energy intake)	17.57	17.39	+0.11	−0.05	+0.20	+0.40
Carbohydrates (% of total energy intake)	50.51	51.22	+1.65 *	+1.84 **	+3.29 *	+2.40 *
Fats (% of total energy intake)	31.87	31.32	−1.79 *	−1.82 *	−3.45 *	−2.76 *
Saturated fats (% of total energy intake)	10.88	10.62	+0.07	+0.04	−0.62 **	−0.43
Free sugars (% of total energy intake)	10.37	10.64	+3.15 *	+3.28 *	+0.70	+0.24
Dietary fibre (g/1000 kcal)	9.50	9.57	−1.86 *	−1.71 *	+0.53	+0.53
Vitamin A (mcg/1000 kcal)	497.77	473.70	−193.20 *	−170.32 *	+9.88	−9.42
Vitamin C (mg/1000 kcal)	54.05	54.56	−10.13 *	−7.51	−0.30	−1.16
Folates (mcg/1000 kcal)	116.18	116.16	−23.02 *	−21.73 *	+2.74	+4.14
Sodium (mg/1000 kcal)	1611.80	1656.68	−189.59 *	−221.96 *	−157.57 *	−105.76
Potassium (mg/1000 kcal)	1680.08	1672.16	−264.47 *	−214.41 *	+236.49 *	+163.26
Calcium (mg/1000 kcal)	462.18	454.18	−32.84 **	−7.05	+179.76 *	+124.46 *
Phosphorus (mg/1000 kcal)	681.58	678.93	−29.14 *	−18.99	+88.80 *	+62.95 *
Magnesium (mg/1000 kcal)	139.24	138.76	−11.80 *	−9.56 *	+16.60 *	+14.66 *
Iron (mg/1000 kcal)	5.63	5.59	−0.48 *	−0.48 *	+0.28	+0.27
Zinc (mg/1000 kcal)	5.12	5.00	+0.09	+0.08	+0.35 *	+0.41 **

^a^ Adjusted for age, sex, parental educational status and typology of neighbourhood; * *p* < 0.01 and ** *p* < 0.05.

## Data Availability

All data generated or analyzed during this study are available from the corresponding author on reasonable request.
